# *Candida* Species Prevalence Profile in HIV Seropositive Patients from a Major Tertiary Care Hospital in New Delhi, India

**DOI:** 10.1155/2016/6204804

**Published:** 2016-03-22

**Authors:** Monika Maheshwari, Ravinder Kaur, Sanjim Chadha

**Affiliations:** ^1^Department of Medical Microbiology, Medeor 24x7 Hospital, P.O. Box 26918, Dubai, UAE; ^2^Department of Microbiology, Lady Hardinge Medical College, New Delhi 110001, India; ^3^Department of Biotechnology & Molecular Diagnostics, National Centre for Disease Control, New Delhi 110054, India

## Abstract

*Candida* is a common opportunistic pathogen during the course of human immunodeficiency virus (HIV) disease progression. Changes in the clinical severity of candidiasis and the* Candida* species prevalence profile may be a reflection of immunological changes in HIV positive patients. The aim of this study was to document the changing pattern of* Candida* species prevalence profile in HIV seropositive patients from a tertiary care hospital in North India. One hundred and twenty HIV seropositive subjects were recruited for* Candida* microbial screening. Clinical specimens including blood, oral swabs, expectorated or induced sputum/bronchoalveolar lavage specimens, and urine were collected depending on the patient's symptoms. A total of 128* Candida* isolates were obtained from 88 cases and 7 different* Candida* species were identified.* C. albicans* (50%) was the most common species isolated followed by* C. glabrata* (17%) and* C. dubliniensis* (12.5%). Other species isolated were* C. parapsilosis* (7.8%),* C. krusei*,* C. tropicalis* (4.6% each), and* C. kefyr* (3%). Strong clinical suspicion along with optimal sampling of an accurate diagnosis of* Candida* species involved would go a long way in decreasing the morbidity associated with non-*albicans Candida* species.

## 1. Introduction

As the AIDS (acquired immunodeficiency syndrome) epidemic enters its fourth decade, HIV transmission in most parts of the world shows no signs of abating despite great progress being made in tackling the epidemic worldwide [1]. It still continues to grow in countries of all incomes with a greater impact on the more vulnerable developing world and has emerged as a global crisis since its discovery in the summer of 1981 [2]. Globally, an estimated 35.3 million people were living with HIV, 2.3 million new people became infected, and 1.6 million deaths occurred in the year 2012 [3].

Owing to a weakened immune system, the infected person is placed at an increased risk of a wide variety of opportunistic infections [4, 5]. A number of protozoa, fungi, viruses, and bacteria are responsible for such infections [6, 7]. Fungal opportunistic infections in patients infected with HIV are a major cause of morbidity and mortality [8] and compromise the quality of life of such individuals. Oral candidiasis has been reported as the most common fungal opportunistic infection by some previous studies [9, 10]. The spectrum of* Candida* infection is diverse, ranging from asymptomatic colonization to oropharyngeal candidiasis (OPC), esophagitis, onychomycosis, vulvovaginitis, cutaneous candidiasis, and systemic candidiasis or invasive candidiasis including candidemia [11]. It has been seen that almost all HIV infected people are colonized with* Candida* and up to 90–95% develop clinical lesions as the viral disease progresses [12]. It has also been observed that low CD4 counts and high plasma HIV RNA levels significantly correlate with oral* Candida* carriage as well as with oral candidiasis in HIV patients [13–15]. These data suggest that a decrease in oropharyngeal* Candida* carriage and oral candidiasis in HIV can be achieved by initiating patients on highly active antiretroviral therapy (HAART) without the need for specific antifungal therapy. Thus a prompt diagnosis of* Candida* infection in HIV patients and assessment of the immune status can have bearing on treatment of such infections and improve the general well-being of the PLWHA (people living with HIV/AIDS).

Though* Candida albicans* is the most frequently isolated species as a colonizer and pathogen of the oral mucosa, other* Candida* species, such as* C. tropicalis*,* C. krusei*,* C. glabrata*,* C. dubliniensis*,* C. guilliermondii*,* C. parapsilosis*,* C. kefyr*, and* C. pelliculosa*, have become a significant cause of infection in patients with HIV infection [16–18]. The clinical importance of these non-*albicans Candida* species lies in the fact that they are usually less susceptible to the more commonly used azole antifungal drugs, a factor that poses significant difficulties in effective treatment [19]. Thus the modern Mycology Laboratory has an important role to play in several aspects relating to these organisms, including detection, identification, epidemiological analysis, and therapy in an attempt to better understand these pathogens and provide an effective cure [20].

In view of the above this study was conducted to determine the prevalence of candidiasis, to identify the various* Candida* species implicated in causation, and to study the relationship of* Candida* infections to the immunological status of HIV positive patients in the biggest tertiary care hospital of New Delhi, India.

## 2. Materials and Methods

### 2.1. Study Population

One hundred and twenty HIV seropositive adult subjects attending the outpatient departments, ART clinic, and those admitted in the various wards of Lok Nayak Hospital affiliated to Maulana Azad Medical College, New Delhi, India, were recruited for this study irrespective of their antiretroviral therapy status for* Candida* microbial screening. Lok Nayak Hospital is a 2500-bedded tertiary care facility with over 3000 patients enrolled in the antiretroviral therapy clinic. The Microbiology Department is a regional sentinel surveillance centre for HIV/AIDS.

Only those HIV seropositive subjects who have not received any specific antifungal therapy in the preceding three months were enrolled. Fifty age and sex matched randomly selected adult HIV seronegative subjects were also enrolled from the Mycology Laboratory of Department of Microbiology, Maulana Azad Medical College, New Delhi.

### 2.2. Study Design

This was a cross-sectional analysis to determine the clinical, immunological, and microbiological* Candida* profile of HIV seropositive cases and HIV seronegative control subjects. At enrolment an informed consent was obtained and each study participant was asked to complete a questionnaire which consisted of sociodemographic and personal details, history of present illness, clinical signs and symptoms, and so forth. Subjects were staged as per WHO staging system which ranges from the asymptomatic stage (stage I) to mild symptoms (stage II), advanced symptoms (stage III), and severe symptoms (stage IV) [21]. The study period was from May 2013 to December 2014.

### 2.3. Sample Collection

Various clinical specimens including blood, oral swabs, expectorated or induced sputum/bronchoalveolar lavage specimens, and urine were collected depending on the patient's clinical presentation as per our established laboratory protocol using standard precautions. All specimens were transported to the Mycology Laboratory as soon as possible without any delay and were processed on the same day of collection.

### 2.4. Laboratory Procedures

Diagnosis of HIV infection was done by following the standard protocol at our Integrated Counselling and Testing Centre that employs pretest and posttest counselling and obtains informed consent before HIV testing. Three different rapid tests were used to detect HIV-1 and HIV-2 antibodies (CombAids (Span Diagnostics Ltd.), Retrocheck HIV (Qualpro Diagnostics), and Tri-Line (Rapid Diagnostics)) following the manufacturer's instructions.

The samples were subjected to direct microscopy using Gram staining, KOH wet mount, and India ink preparations depending on the type of specimen. Fungal culture was done on Sabouraud dextrose agar, with and without chloramphenicol (16 *μ*g/mL) and with cycloheximide (0.3 *μ*g/mL) plus chloramphenicol (16 *μ*g/mL), brain heart infusion agar, and 5% sheep blood agar. Specimens were streaked in duplicate; one set of inoculated slants was incubated at 25°C and the other was incubated at 37°C, and they were examined every other day for growth up to 4–6 weeks before discarding as negative. Samples inoculated on blood agar were incubated for 24–48 h and samples on brain heart infusion agar were incubated for 1-2 weeks. Fungal growth was identified by colony morphology, Gram staining, lacto phenol cotton blue preparation, and Riddle's slide culture as per standard recommended procedures. Identification and speciation of yeast isolates were done on the basis of germ tube production, morphology on corn meal agar with teen 80, HiCrome* Candida* agar, carbohydrate fermentation test, carbohydrate assimilation test, morphology on Staib, and Caffeic Acid Ferric Citrate agar as per standard recommended procedures [22–24].

The CD4 T lymphocyte count of all participants was determined by the FACSCount system by Becton Dickinson. This instrument works on the principle of Flow Cytometry (single platform technology) [25].

### 2.5. Statistical Analysis

Data were statistically described in terms of frequencies and percentages and 95% confidence interval was calculated for all prevalence estimations. Continuous quantitative variables like CD4 counts with an approximately normal distribution were analyzed by calculating the mean, standard deviation, and 95% confidence interval by Student's paired *t*-test. Means of categorical data were compared using the one-way ANOVA test. The adopted significance level for statistical inference was 5%. All statistical calculations were done using computer program Statistical Package for the Social Sciences (SPSS; SPSS Inc., Chicago, IL, USA) version 15 for Microsoft Windows.

## 3. Results

Out of the 120 study participants recruited, there were 82 males and 38 females. The sociodemographic profile of our participants and the control group is shown in Table 1. Ninety-four percent of the study population belonged to the age group of 21–45 years which is the sexually active population group. The age range of our study participants was 18–55 years. Most of our patients hailed from Delhi and the neighbouring states of North India. Out of all the study subjects investigated majority were illiterate or had received education up to high school.

Table 2 shows the clinical profile of our study subjects. Fever, weight loss, and cough were the most common symptoms in HIV seropositive cases. White oral patches, oral ulcers, burning micturition, and painful swallowing were seen in 28%, 26%, 20%, and 13% of patients, respectively. Majority of our patients (72%) belonged to WHO clinical stage III or stage IV.

Table 3 shows the immunological profile of our study subjects. The mean CD4 T lymphocyte count of our HIV seropositive study participants was 272.3 cells/*μ*L at the time of recruitment. The CD4 counts ranged from 25 to 758 cells/*μ*L with 17% exhibiting severe immunosuppression with CD4 counts of less than 100 cells/*μ*L. Patients with single complaint, double complaint, three complaints, and four complaints had significantly higher CD4 counts when compared individually to patients with five complaints (mean and standard deviations are shown in Table 3; *p* values < 0.05). Subjects in whom* Candida* infection was detected had significantly lower CD4 counts as compared to subjects in whom* Candida* infection was not detected (*p* value < 0.05). Patients in WHO clinical stage I, stage II, and stage III had significantly higher CD4 counts as compared to patients in WHO clinical stage IV (*p* value < 0.05). Out of the 120 HIV seropositive study participants, 42 patients (43%) were on HAART, out of which 34 patients were on first-line therapy (Zidovudine + Lamivudine + Nevirapine) and 8 patients were on second-line therapy (Stavudine + Lamivudine + Nevirapine), 4 of which had active pulmonary tuberculosis and four exhibited Zidovudine induced anaemia after being on first-line therapy. The mean duration of therapy for our patients was 10 months 6 days.

A total of 352 samples were collected from 120 patients.* Candida* species were isolated in 128 samples from 88 cases, most often from oral swabs (82 out of 120 collected) followed by sputum and urine specimens, as shown in Table 4. Based on germ tube test, morphology on corn meal agar and HiCrome agar, carbohydrate fermentation, and carbohydrate assimilation test* C. albicans* (50%) was the most common species isolated followed by* C. glabrata* (17%).* C. dubliniensis* (12.5%) was identified on the basis of colony morphology and chlamydospore formation on Niger seed agar, Caffeic Acid Ferric Citrate agar, and heat test at 45°C. Other species isolated were* C. parapsilosis* (7.8%),* C. krusei*,* C. tropicalis* (4.6% each), and* C. kefyr* (3%). Out of 64* C. albicans* strains, the maximum number (38/64) was from oral swabs followed by sputum (18/64) and urine (6/64), while most of* C. glabrata* strains (12/22) were isolated from urine samples.* C. dubliniensis*,* C. parapsilosis*,* C. krusei*,* C. tropicalis*, and* C. kefyr* were mainly detected in oral swabs. In 40/120 cases we detected 2 different* Candida* isolates but not from the same clinical specimen.

Oral swabs from 60 age and sex matched HIV seronegative healthy adults were processed and* Candida albicans* was isolated from 17 cases by using the identification methods mentioned above.

## 4. Discussion

*Candida* species are ubiquitous fungi causing severe disease in immunocompromised individuals like AIDS patients with extremely varied clinical manifestations. They may be localized to the mouth, lungs, or the gastrointestinal tract or may become systemic as in septicaemia, endocarditis, and meningitis. Management of candidiasis is hampered by delays in diagnosis and lack of reliable diagnostic methods which allow detection up to the species level. To assist the clinician in therapeutic decisions and optimize treatment, clinical microbiologists should identify* Candida* to species level especially in HIV positive patients in whom non-*albicans Candida* species are being increasingly recognized to cause serious infections. With this view in mind this study was conducted to establish the profile of* Candida* infections in HIV positive patients and to correlate it with their immunological status as there is paucity of such data from the Indian HIV seropositive population.

The mean age of HIV seropositive subjects in our study was 33.4 ± 6.2 years with an age range of 18–55 years and the age group of 21–45 years was the most commonly affected age group. There was male preponderance observed in our study with a male-to-female ratio of 2.2 : 1. This correlates well with the demographic variables of the HIV positive population in the country and highlights the fact that males are at increased risk in comparison to females because of their jobs and habits which entail them to be more migratory in comparison to females. A few other investigators who studied the* Candida* profile and spectrum of opportunistic infections in HIV positive patients have also reported similar findings [11, 26].

The most common clinical presenting complaints of our HIV seropositive study participants were fever (70%), weight loss (50%), and cough (47%). These clinical symptoms of the HIV positive patients from our hospital are representative of the clinical picture of HIV/AIDS in the country and are very similar to what has been reported by researchers across other parts of India [27, 28]. Presence of white oral patches (28%), oral ulcers (26%), and painful swallowing (13%) seen in our study patients constitute clinical features of oral thrush and esophageal candidiasis. These contribute to poor oral intake and weight loss and significantly affect the quality of life of such patients and are considered important markers of clinical disease progression and immunosuppression [9]. Burning micturition seen in 30% of our patients may have been due to urinary candidiasis. As seen with other studies [10, 29], majority (79.9%) of our HIV positive patients presented with more than one clinical symptom and 72% of our patients presented with WHO clinical stage III or stage IV events at the time of recruitment.

The mean CD4 T lymphocyte count of our HIV seropositive study population at recruitment was 272.3 cells/*μ*L with a range varying from 25 to 758 cells/*μ*L. Similar results have been reported by another researcher by his investigations on HIV infected patients in China, wherein the average CD4 cell count was 253.4 cells/mm^3^, with a range from 2 to 1050 cells/mm^3^ in all patients [30]. In the present study the mean CD4 T lymphocyte count in 88 patients with* Candida* infection was 142.6 cells/*μ*L, with a range of 25–463 cells/*μ*L and a standard deviation of ±72. This was found to be significantly lower than the mean CD4 counts of 32 patients without* Candida* infection (412.3 cells/*μ*L; range of 256–583 cells/*μ*L and standard deviation of 93; *p* value < 0.05). This shows that cell mediated immune response is an essential host defence against candidiasis. This observation has been well supported by other investigators who have reported a strong correlation of the occurrence of candidiasis in HIV positive patients with lower CD4 counts [30–32]. This also reinforces the 2013 WHO recommendations to initiate treatment in adults living with HIV when their CD4 cell count falls to 500 cells/mm^3^ or less in order to maximize the drug benefits and prevent* Candida* related morbidity in PLWHA.

In our study oral swabs yielded the highest number of* Candida* isolates followed by sputum and urine specimens. Blood culture specimens exhibited the lowest yield for* Candida* species. These observations have been documented by a few other studies which have also reported the highest yield of* Candida* spp. from oral swabs and the lowest yield of* Candida* spp. from blood specimens from HIV positive patients [9, 11]. Irrespective of the ART status and the type of specimen processed* Candida albicans* (50% of the total isolates) was the predominant species recovered in our study. This high isolation rate of* Candida albicans* in HIV positive patients is consistent with many previous published reports from India as well as abroad [9, 31, 33, 34]. The most common non-*albicans Candida* species identified in our study population were* C. glabrata* (17.1% of the total isolates) and* C. dubliniensis* (12.5% of the total isolates).  Table 5 shows the distribution of* Candida* species isolated from HIV positive patients in India in the last few years by various investigators. The higher detection rate of* C. dubliniensis* in our study may be due to the fact that this species was previously misidentified due to its phenotypic resemblance to* C. albicans* and it is now being increasingly recognized.

Another important finding of our study was that the prevalence of* Candida* species was significantly higher (*p* value < 0.05) in the HIV positive study group (88 cases out of 120 recruited; 73.3%) as compared to the HIV negative control group (17 cases out of 60 recruited; 28.3%), similar to the results of other studies [30, 31]. No non-*albicans Candida* species were isolated in the HIV seronegative control group.

This study had a few limitations. Since the sample size in the present study was small, further studies with a larger sample size are required to substantiate the results. The lack of use of PCR based genotypic methods for identification of* Candida* species may have resulted in some inaccuracies and misdiagnosis of the* Candida* species. We have not reported here the fungal pathogens other than* Candida* spp. isolated from the clinical samples as this study pertains to the prevalence profile of* Candida* spp. only. The clinical symptoms reported by patients could have been due to other fungal as well as nonfungal pathogens.

## 5. Conclusions

Our study gives an insight into the present scenario of* Candida* species prevalence profile of the HIV positive population from New Delhi's busiest and largest tertiary care hospital.* Candida* species are more frequently isolated in the HIV seropositive population and the CD4 T lymphocyte counts of patients with* Candida* infection are significantly lower than those of individuals without* Candida* infection. The proportion of* Candida* infections caused by* C. albicans* in HIV seropositive individuals has fallen and there is a shift in the distribution of* Candida* species in HIV positive patients towards the non-*albicans Candida* species. These latter species tend to be less susceptible to antifungal agents and this has accounted for their emergence as a significant pathogen. Recurrent fungal infections due to the immunocompromised state and repeated exposure to antifungal agents are responsible for this shift in the spectrum in HIV positive cases. The use of accurate and reliable diagnostic methods which readily identify the non-*albicans* species could assist the clinicians in making the right therapeutic choices and check the emergence of antifungal resistant strains. Also reserving antifungal drug prophylaxis for only those with severe and frequent recurrences of candidiasis in HIV patients can go a long way in avoiding antifungal resistance.

## Figures and Tables

**Table 1:** Sociodemographic profile of study subjects.

Patient information	HIV seropositive study subjects	HIV seronegative control subjects
	*n* = 120	*n* = 50
Age		
Mean age ± SD	33.4 ± 6.2	32.1 ± 4.8
Gender, number (%)		
Male	82 (68)	36 (72)
Female	38 (32)	14 (28)
M/F ratio	2.2	2.5
State-wise distribution, number (%)		
Delhi	58 (48)	40 (80)
Uttar Pradesh	46 (38)	8 (16)
Haryana	14 (12)	2 (4)
Madhya Pradesh	2 (2)	0
Educational status, number (%)		
Illiterate	50 (41.6)	20 (40)
Primary school	26 (21.6)	14 (28)
High school	26 (21.6)	8 (16)
Graduate	16 (13.3)	6 (12)
Postgraduate	2 (1.7)	2 (4)

**Table 2:** Clinical profile of study subjects. *n* = 120.

		Number	Percentage
Presenting complaints	Fever	84	70
	Weight loss	60	50
	Cough	56	47
	White oral patches	34	28
	Oral ulcers	32	26
	Burning micturition	24	20
	Painful swallowing	16	13
	Skin lesions	2	2
	Dyspnoea/chest pain	4	3
	Diarrhoea	6	5
	Asymptomatic	16	13

Single/multiple complaints	Single complaint	8	6.5
	Double complaint	34	28.3
	Three complaints	46	38.3
	Four complaints	12	10
	Five complaints	4	3.3

WHO clinical staging	Stage I	16	13
	Stage II	18	15
	Stage III	48	40
	Stage IV	38	32

**Table 3:** Immunological profile of study subjects. *n* = 120.

		Number	Percentage	
CD4 count (cells/*µ*L)	0–50	6	5%	
	51–100	14	12%	
	101–200	26	22%	
	201–300	28	23%	
	301–400	32	27%	
	401–500	6	5%	
	>500	8	7%	

		Mean (standard deviation)	95% confidence interval	*p* value

Mean CD4 counts in relation to the presenting complaints (cells/*µ*L)	Single complaint *n* = 8	318 (86)	176 to 389	0.0004
	Double complaint *n* = 36	272.1 (118)	202 to 344	0.0001
	Three complaints *n* = 48	223 (107)	179 to 268	0.0001
	Four complaints *n* = 12	136 (94)	21 to 251	0.0045
	Five complaints *n* = 4	49 (22)	21 to 109	

		Mean (standard deviation)	95% confidence interval	*p* value

Mean CD4 counts in relation to the presence of *Candida* infection (cells/*µ*L)	Subjects with *Candida* infection *n* = 88	142.6 (72)	68 to 201	0.0001
	Subjects without *Candida* infection *n* = 32	412.3 (93)	189 to 516	

		Mean (standard deviation)	95% confidence interval	*p* value

Mean CD4 counts in relation to WHO clinical stage (cells/*µ*L)	Stage I *n* = 16	407.8 (114)	312 to 502	0.0001
	Stage II *n* = 48	308.2 (98)	218 to 489	0.0001
	Stage III *n* = 18	190.7 (92)	95 to 263	0.0001
	Stage IV *n* = 38	96.1 (45)	35 to 116	

**Table 4:** Sample distribution and *Candida* profile of study subjects. *n* = 120.

		Number	Percentage	
Type of specimens collected (*n* = 352)	Oral swabs	120	34	
	Sputum	56	15.9	
	Urine	80	22.7	
	Blood	96	27.3	

		Direct microscopy, number (%)	Culture, number ( %)	

Different clinical specimens showing *Candida* isolates (*n* = 352)	Oral swabs *n* = 120	38 (31.6)	82 (68.3)	
	Sputum *n* = 56	14 (25)	26 (46.4)	
	Urine *n* = 80	16 (20)	18 (22.5)	
	Blood *n* = 96	0	2 (2.1)	

		Number	Percentage	95% CI of prevalence

*Candida* species isolated (*n* = 128)	*C. albicans*	64	50	0.414, 0.585
	*C. glabrata*	22	17.1	0.116, 0.246
	*C. dubliniensis*	16	12.5	0.078, 0.193
	*C. parapsilosis*	10	7.8	0.043, 0.137
	*C. krusei*	6	4.6	0.021, 0.098
	*C. tropicalis*	6	4.6	0.021, 0.098
	*C. kefyr*	4	3.1	0.012, 0.077

		Number	Percentage	

Number of *Candida* isolates in recruited cases (*n* = 120)	Single isolate	48	40	
	Double isolate	40	33.4	
	No isolate	32	26.7	

**Table 5:** 

Year	Place of study/researcher	Patient characters	Predominant *Candida* species	95% CI of prevalence	Reference number
2001	Chennai (Menon et al.)	46 *Candida* isolates from HIV positive patients	*C. albicans* (73.9%)	0.61 to 0.86	[35]
			*C. tropicalis* (21.7%)	0.14 to 0.39	
			*C. dubliniensis* (0%)		

2004	Delhi (Lattif et al.)	75 *Candida* isolates from HIV positive patients	*C. albicans* (86%)	0.76 to 0.92	[36]
			*C. parapsilosis* (8%)	0.04 to 0.16	
			*C. glabrata* (3%)	0.01 to 0.09	
			*C. krusei* (3%)	0.01 to 0.09	
			*C. dubliniensis* (0%)		

2009	Mumbai (Baradkar and Kumar)	50 *Candida* isolates from HIV positive patients	*C. albicans* (70%)	0.56 to 0.81	[37]
			*C. parapsilosis* (15%)	0.07 to 0.26	
			*C. glabrata* (7.5%)	0.04 to 0.19	
			*C. tropicalis* (5%)	0.02 to 0.16	
			*C. dubliniensis* (2.5%)	0.003 to 0.105	

2013	Lucknow (Maurya et al.)	84 *Candida* isolates from HIV positive patients	*C. albicans* (95.2%)	0.88 to 0.98	[31]
			*C. tropicalis* (4.76%)	0.02 to 0.12	
			*C. dubliniensis* (0%)		

## Additional Points

The research was performed at Department of Microbiology, Maulana Azad Medical College, and Lok Nayak Hospital, Bahadur Shah Zafar Marg, New Delhi, India.
